# Lithium ion storage between graphenes

**DOI:** 10.1186/1556-276X-6-203

**Published:** 2011-03-09

**Authors:** Yue Chan, James M Hill

**Affiliations:** 1 Nanomechanics Group, School of Mathematical Sciences, The University of Adelaide, Adelaide, SA 5005, Australia

## Abstract

In this article, we investigate the storage of lithium ions between two parallel graphene sheets using the continuous approximation and the 6-12 Lennard-Jones potential. The continuous approximation assumes that the carbon atoms can be replaced by a uniform distribution across the surface of the graphene sheets so that the total interaction potential can be approximated by performing surface integrations. The number of ion layers determines the major storage characteristics of the battery, and our results show three distinct ionic configurations, namely single, double, and triple ion forming layers between graphenes. The number densities of lithium ions between the two graphenes are estimated from existing semi-empirical molecular orbital calculations, and the graphene sheets giving rise to the triple ion layers admit the largest storage capacity at all temperatures, followed by a marginal decrease of storage capacity for the case of double ion layers. These two configurations exceed the maximum theoretical storage capacity of graphite. Further, on taking into account the charge-discharge property, the double ion layers are the most preferable choice for enhanced lithium storage. Although the single ion layer provides the least charge storage, it turns out to be the most stable configuration at all temperatures. One application of the present study is for the design of future high energy density alkali batteries using graphene sheets as anodes for which an analytical formulation might greatly facilitate rapid computational results.

## Introduction

The development of an efficient lithium ion battery, which has the highest energy density and the quickest recharge time, relies on a complicated optimization of novel materials for the anode, the cathode, and the electrolyte. Graphite is currently the most common material used for the anodes of commercial batteries because of its capability for reversible lithium intercalation in the layered crystals, which represents the maximum theoretical lithium storage capacity, around 372 mAh/g [1]. A single layer of graphite, referred to as graphene, has been synthesized using the mechanical exfoliation of graphite by Novoselov et al. [2], and quite recently the 2010 Nobel Prize for Physics was awarded to A. Geim and K. Novoselov for this discovery. The extreme mechanical and chemical properties of graphene have already been exploited for possible energy storage and microelectronics [3,4]. Numerous experiments have been performed to confirm the utilization of graphene nanosheets and nanoribbons to enhance lithium storage capacity and to improve recharge cyclic performance [5-7]. Semi-empirical molecular orbital calculations have been used to investigate lithium ion storage states between two graphene sheets [8], as well as some heteroatom-substituted carbon materials [9]. Density functional theory has also been used to investigate the structure, bonding, and magnetic properties of metal atoms embedded between graphenes [10]. Other hybrid carbon structures containing graphenes such as silicon-graphene [11], TiO-graphene [12], and Sn-graphene [13] have been shown experimentally to possess very high ion storage capacities.

In this article, we adopt the continuous approach employed by Cox et al. [14,15] and the 6-12 Lennard-Jones potential and we assume that the carbon atoms can be uniformly distributed across the surface of nano-structures, so that the total potential energy between various non-bonded molecules can be determined analytically by performing surface integrations. The total potential energy can be used to investigate the relative motion of certain nano-structures, such as the oscillatory motion of a fullerene or an ultra-small nanotube inside a single-walled carbon nanotube [15]. In addition, the same methodology has successfully been used to study the encapsulation of drug molecules inside single-walled nanotubes as the 'magic bullet' concept [16,17] and the encapsulation of methane molecules and hydrogen atoms inside metal-organic frameworks for gas storage [18].

**Figure 1:**
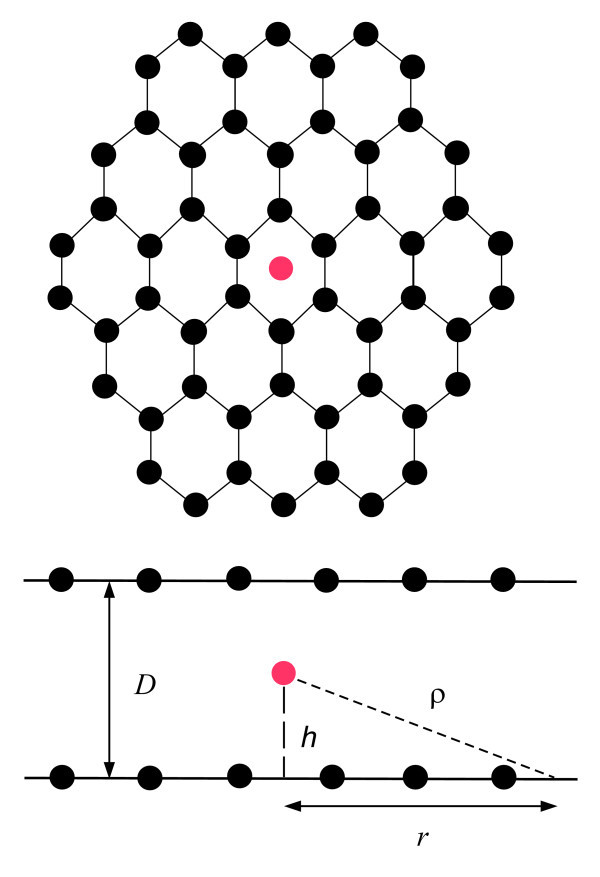
**Lithium ion stored between two parallel graphene sheets**.

The present authors have investigated the minimum molecular spacing between two parallel graphene sheets for stable atom/ion storage, and we have determined the diffusion time for atom/ion leaving the graphene sheet of a given size under different temperatures [19]. Here, we investigate lithium ion storage between two parallel graphene sheets. The continuous approach is employed to approximate the van der Waals interaction between a single lithium ion and the graphene sheets, so that the equilibrium positions of the lithium ion between the graphene sheets *h *can be determined for a given separation *D *(see Figure 1), from which, we can deduce the number of possible ion layers that might be formed between two graphenes. Three distinct ion layers, namely single, double and triple layers for *D *= 5, 7.7, and 8.3 Å are predicted. While the double and triple ion layers are found to provide storage capacities exceeding that of conventional graphitic carbon materials [1,8], the single ion layer is found to provide the most stable option for ion batteries operating under extreme temperatures. Wherever possible, we compare our theoretical results with those obtained by Suzuki et al. [8] using semi-empirical molecular orbital calculations.

In the next section, we present the continuous approach in the context of the current investigation. Numerical results and discussions are given in "Numerical results and discussion" section and a general conclusion is provided in the final section.

## Theory

In this section, we summarize the theoretical background for the article. We model the interaction between a lithium ion and a graphene sheet using the non-bonded Lennard-Jones potential. During the charging process, the system appears to be neutral [8] and therefore we may use the Lennard-Jones potential to determine the molecular interaction energy. The 6-12 Lennard-Jones potential *V*(*ρ*) [20] for two non-bonded atoms is given by(1)

where *ρ*, *ε*, and *σ *denote the atomic distance between the two atoms, the potential well depth of two atoms, and the Lennard-Jones distance between two atoms, respectively. In addition, *A *and *B *denote the attractive and repulsive Hamaker constants, respectively. We comment that following the description by Jones [20,21], many theoretical efforts have been attempted to improve Jones's empirical results by taking into account the dielectric properties of the molecular surface [22]. Next, we assume that the carbon atoms are smeared across the surface of the graphene sheet so that the continuous approximation used by Cox et al. [14,15] can be employed to determine the total energy between the single lithium ion and the single graphene sheet, which can be written as(2)

where eta and *dS *denote the atomic number density, i.e., number of carbon atoms per unit area and the surface area element of the graphene sheet, respectively. We comment that it is analytically convenient to approximate the graphene sheet by a circular shape, for which we can take *dS *= *2πrdr*. Upon computing *E*_1/2_, the total energy of the lithium ion between two parallel graphenes *E *can be determined by the sum of the total energy arising from the upper and lower graphene sheets. The schematic diagram for the proposed system is shown in Figure 1. Under these assumptions, the total energy of the system becomes(3)

where *h*, *D*, and *r *denote the perpendicular distance between the ion and the lower graphene sheet, the separation between the two graphenes, and the radial distance in the planes of the graphene sheets, respectively. In addition, we assume that the temperature-dependent number density of lithium ions between two graphenes can be written as(4)

where *T*, *n*_0_, and *k*_B _denote the temperature, the number density at absolute zero, and the Boltzmann's constant, respectively. We comment that 1 - exp(-|*E*|/*k*_B_*T*) represents the probability that the ion will exit the region between the graphene sheets at temperature *T *[18]. The total number of ions stored between the graphene sheets for different temperatures is given by(5)

where *V *denotes the cavity volume between the two graphenes. Equation (5) can be readily evaluated using a numerical integration technique such as Simpson's Rule.

## Numerical results and discussion

**Figure 2:**
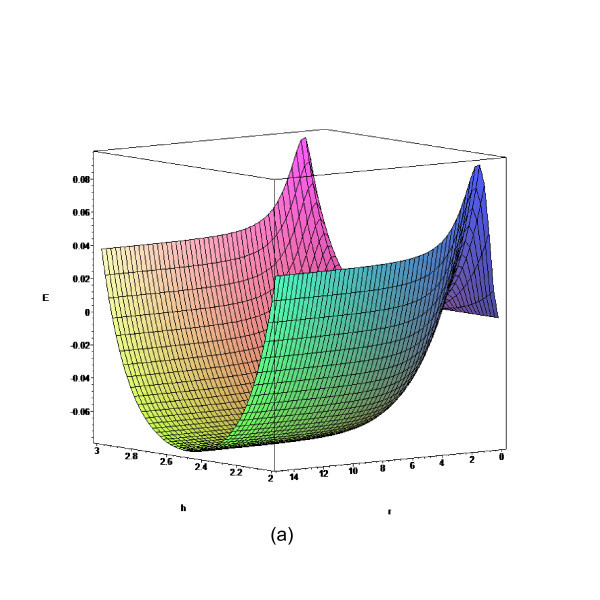
**Total energy for lithium ion stored between two parallel graphene sheets with *D *= 5 Å**.

**Figure 3:**
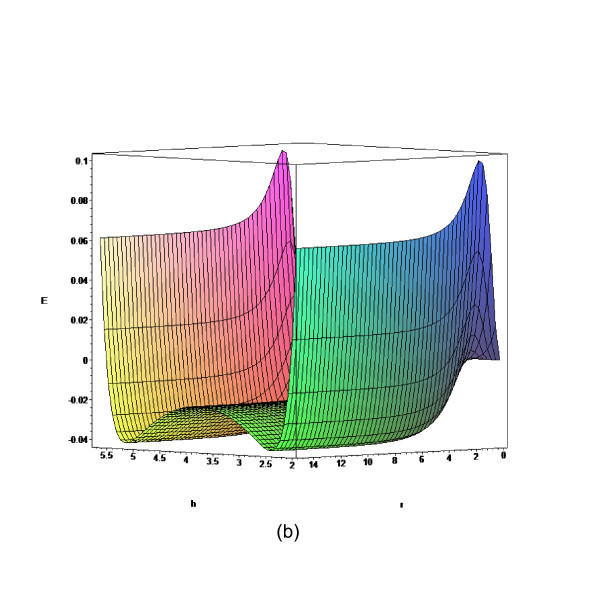
**Total energy for lithium ion stored between two parallel graphene sheets with *D *= 7.7 Å**.

**Figure 4:**
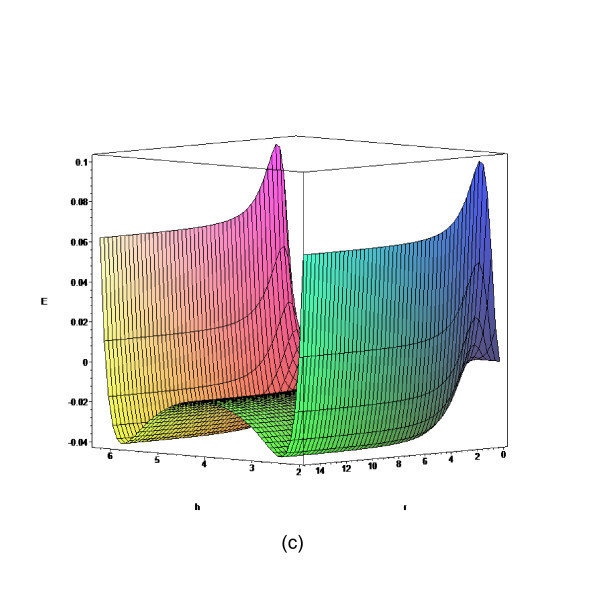
**Total energy for lithium ion stored between two parallel graphene sheets with *D *= 8.3 Å**.

**Figure 5:**
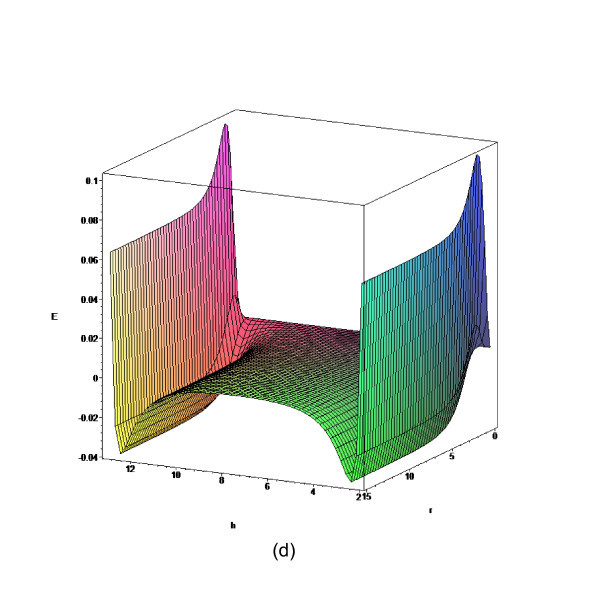
**Total energy for lithium ion stored between two parallel graphene sheets with *D *= 15 Å**.

In this section, we obtain some numerical results according to the formulae given in "Theory" section. Three separation distances, namely, *D *= 5, 7.7, and 8.3 Å are investigated due to the fact that they allow a single layer, a double layer, and a triple layer of lithium ions embedded between two parallel graphenes, respectively [8]. The total energy for a lithium ion embedded between two graphene sheets is determined from Equation (3) and the numerical values for *E *for the prescribed values of *D *are shown in Figures 2, 3, 4 and 5. The numerical values of the parameters *A*, *B*, and eta are given in Table 1.

**Table 1:** Parameters for proposed systems (*A *and *B *are determined by the Lorentz-Berthelot mixing rule [24] and layer densities from [8])

Description	Parameter	Value
Attractive constant	*A*	3.959 eVÅ^6^
Repulsive constant	*B*	904.438 eVÅ^12^
Number density (graphene)	eta	0.381 Å^-2^
Number density (single layer)	*n*_1_	0.123 Å^-2^
Number density (double layer)	*n*_2_	0.147 Å^-3^
Number density (triple layer)	*n*_3_	0.180 Å^-3^

**Figure 6:**
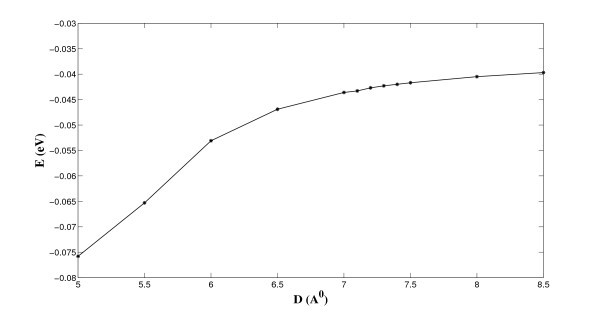
**Minimum energy versus *D *for *r *= 10 Å**.

We observe from Figure 2 that there is a single minimum for *D *= 5 Å, while from Figures 3 and 4, there are two minima for the cases *D *= 7.7 and *D *= 8.3 Å, respectively. The central plateau for *D *= 8.3 Å is flat enough to accommodate more lithium ions, which corresponds to the double ion and triple ion layers for *D *= 7.7 and *D *= 8.3 Å, respectively. As a benchmark, we also plot the total energy for *D *= 15 Å in Figure 5, and we observe that the central plateau widens to accommodate more ion layers than that for *D *= 15 Å. The numerical results agree well with similar results obtained using the semi-empirical molecular orbital calculations [8]. We also note that the total energy asymptotically approaches a certain value when the radial distance is sufficiently large, which demonstrates the rapid decay of the Lennard-Jones potential at larger distances [23]. Next, we fix *r *= 10 Å and vary *h *to investigate the minimum potential energy versus the separation *D*, which is shown in Figure 6.

**Figure 7:**
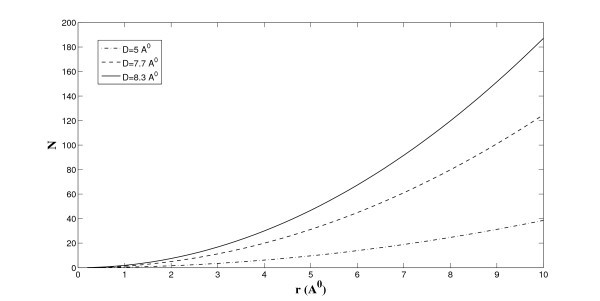
**Number of lithium ions stored between graphenes at *T *= 0 K**.

As the separation between two graphene sheets increases, the binding energy between the lithium and the two parallel plates decreases and asymptotically approaches to that of the single graphene sheet. This finding also agrees well with Suzuki et al. [8], for which they measure the absolute value of the binding energy for more than one lithium ion. Next, we relax the constraint of the ion number applied by Suzuki et al. [8] and investigate the total number of lithium ions that could be stored between the graphene sheets of arbitrary size at absolute zero. On letting *T *= 0 K and using Equation (5) and we estimate the number density of lithium for the particular ion layers formed between two parallel plates, which are also given in Table 1. We comment that the semi-empirical molecular orbital calculations provide a more accurate number density estimation than the current simple model. The total number of lithium ions stored between the three proposed distinct configurations for various graphene sizes can be determined, and is shown in Figure 7.

The resulting lithium storage for *r *= 2.8 Å roughly corresponds to that predicted for C54H18 in Suzuki et al. [8]. We comment that the larger the graphene sheets, the more lithium ions can be stored and which becomes beyond the computational capacity of the method of Suzuki et al. [8]. This study might offer a far more realistic engineering estimation procedure for battery design using graphene nanosheets as anodes. The merit of the continuous approximation is that we may predict the lithium storage capacity for graphene sheets of any size, which could be computationally challenging using molecular orbital calculations. We also comment from Figure 7 that the increase in layer numbers involves higher lithium ion storage. For the double and triple ion layers, the calculated storage capacities are both higher than the maximum theoretical storage capacity for conventional graphitic carbon materials, i.e., 372 mAh/g [1], which is approximately equivalent to the case of a single ion layer, i.e., *D *= 5 Å [8]. Although the triple layers store more lithium ions than the double layers, the more sophisticated calculations performed by Suzuki et al. [8] show that the double ion layers configuration is the most preferable candidate for higher lithium storage. Their calculations take into account the charge-discharge property to prevent the formation of hysteresis resulting from a decrease in positive charges of lithium ions with an increase in ion layers.

**Figure 8:**
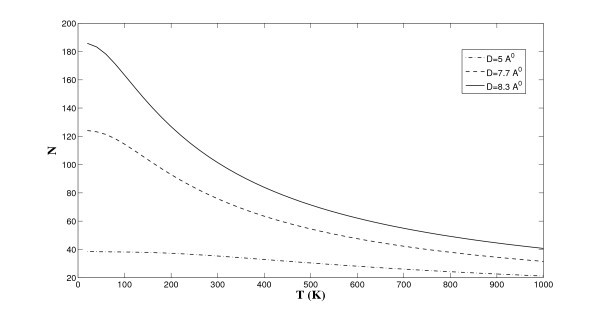
**Number of lithium ions stored between graphenes under different temperatures**.

Next, we incorporate the temperature effect into our model. We fix *r *= 10 Å and use Equation (5) to determine the variation of the lithium storage as a function of the surrounding temperature, and the numerical results are shown in Figure 8. The major merit of our theoretical approach is the rapid computation of the lithium storage under different temperatures, which is entirely ignored in Suzuki et al. [8]. We comment that in all scenarios, the storage capacity decreases due to the leakage of lithium ions as the temperature increases. However, the deeper potential well depth for the single ion layer (see Figure 6) minimizes the rate of ion leakage for the case of the single layer in comparison to that of the double and triple layers. This shows that the double ion layers are preferable for larger storage capacity than those of the conventional graphite or of the single ion layer. If, however, we intend to fabricate a stabler and safer battery system operating at diverse temperatures rather than emphasizing the storage capacity, the single layer ion structure turns out to be the most ideal choice for the battery design up to the maximum storage capacity provided by the current graphite anode.

**Figure 9:**
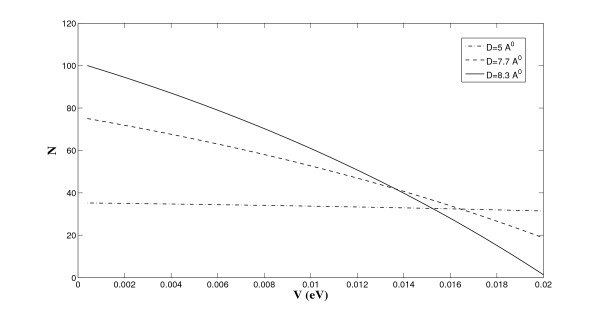
**Number of lithium ions stored between graphenes under an additional external energy**.

External effects arising for example from either pressure, electrical, or magnetic fields will modify the potential landscape of the proposed physical system and hence vary the number of lithium ions stored between graphenes. We may capture such effects by adding an additional homogeneous energy term *V *into Equation (3). We fix *T *= 300 K and *r *= 10 Å to determine the variation of the lithium storage as a function of this external energy *V*, and the numerical results are shown in Figure 9. We comment that in all scenarios, ion storages for three different layer configurations reduce from the corresponding results in Figure 8 at *T *= 300 K, and the storage capacity decreases due to the presence of a positive external energy. Again, the deeper potential well depth for the single ion layer minimizes the rate of ion leakage for the case of the single layer in comparison to that of the double and triple layers. Rather surprisingly, the storage capacity of the double layers surpasses that of the triple layers when *V *exceeds 0.0135 eV, which might easily arise during a charging process. This outcome strengthens the adoption of using double layers as the ideal ion storage configuration.

## Conclusion

In this article, we adopt the continuous approximation and basic statistical mechanics to investigate suitable storage configurations for different battery designs using graphene sheets as the anode. Although we extract some accurate parameters from the molecular orbital calculations, our theoretical methodology yields very rapidly the numerical results for graphene sheets of various sizes under different surrounding temperatures and external effects. While the double layer configuration predicts a larger storage capacity than that of graphite, the single layer configuration turns out to be the most suitable candidate for the safest and stablest ion battery operating at extreme temperatures.

## Competing interests

The authors declare that they have no competing interests.

## Authors' contributions

YC carried out the theoretical studies and drafted the manuscript. JH guided the progress, and improved the presentation and analysis of the manuscript. All authors read and approved the final manuscript.
